# Treatment strategy for BVO-ME based on long-term outcomes correlating retinal structure by OCT image and visual acuity

**DOI:** 10.1186/s12886-023-03138-2

**Published:** 2023-09-20

**Authors:** Yuki Hattori, Ryo Fujiwara, Hidetsugu Mori, Motoki Kimura, Haruhiko Yamada, Kanji Takahashi

**Affiliations:** Department of Ophthalmology, Kansai Medical University, 2-5-1 Shinmachi, Hirakata, Osaka 573-1191 Japan

**Keywords:** Intravitreal anti-VEGF treatment, BRVO, CME, EZ, Foveal bulge, SRD

## Abstract

**Background:**

Intravitreal anti-vascular endothelial growth factor (VEGF) is a mainstream treatment for reducing ME secondary to BRVO (BVO-ME). Regrettably, most reports of intravitreal anti-VEGF for BVO-ME have disclosed only short-term outcomes. Here, we characterized long-term indicators for the visual prognosis of patients with BVO-ME, including the correlation between retinal structure by OCT and visual acuity.

**Methods:**

Patients with BVO-ME were retrospectively recruited based on clinical records in Kansai Medical University Hospital from June 2012 to March 2022. This study enrolled patients with vision loss who received intravitreal injection of anti-VEGF for BVO-ME. Inclusion criteria were that patients received intravitreal injection of anti-VEGF as their first treatment and were followed for at least 36 months. Exclusion criteria were those patients with ocular disease other than BRVO or who had been previously treated for BVO-ME. Patients were divided into two groups according to BCVA at the final visit: Group A (≥ 0.7) and Group B (< 0.7).

**Results:**

Forty-seven eyes from 45 patients were assessed. The mean follow-up period from initial to final visit was 64.38 ± 15.07 (range, 38–100) months. BCVA in Group A (*n* = 32) was significantly greater than in Group B (*n* = 15) at all timepoints. The ratio that the number of eyes which the EZ band and the foveal bulge were intact in Group A was higher than in Group B (*p* = 0.0004 and *p* = 0.0002, respectively). The ratio that the number of eyes which recurrence SRD was observed by the final visit in Group A was lower than in Group B (*p* = 0.0485).

**Conclusions:**

The integrity of the EZ band and an intact foveal bulge were significant predictors for visual acuity. In contrast, recurrent SRD led to poor visual acuity in the long term, even if BCVA was good in the short term.

## Background

Retinal vein occlusion (RVO) is a very common disorder in middle-aged and elderly people with an estimated prevalence of over 16 million worldwide [1]. RVO caused by luminal obstruction of retinal venous outflow, often occurring in patients who have lifestyle-related diseases such as arteriosclerosis, hypertension, hyperlipidemia, and/or diabetes mellitus. RVO is classified based on the site of RVO: central RVO (CRVO), hemi-RVO, and branch RVO (BRVO). The incidence of BRVO is several-fold higher than in those with CRVO or hemi-RVO [2].

Fluorescein angiography (FA), optical coherence tomography (OCT), and OCT angiography (OCTA) have been the primary evaluation tools used to assess the vascular status of RVO. Since severe systemic complications like anaphylactic shock can occur following intravenous administration of fluorescent agents, as done in FA, OCTA has come to play a central role in evaluating the severity of RVO as it can reveal the retinal structure in as much detail as is obtained by FA, but in a less invasive and easier manner. Therefore, the introduction of OCT and OCTA allows us not only to evaluate the vascular network, but also to assess various retinal tissue parameters. This knowledge obtained through OCT and OCTA has contributed to the field’s understanding of BRVO, and many previous clinical reports on this topic have been published.

Since macular edema (ME) is the key factor reducing the patient’s visual acuity due to RVO, to reduce ME secondary to BRVO (BVO-ME) is our clinical entity. Several methods have been employed in an attempt to improve BVO-ME: vitrectomy, administration of drugs (i.e., steroids or inhibitors of vascular endothelial growth factor [VEGF]), and laser photocoagulation. Previous reports demonstrated elevated secretion of VEGF in patients with BRVO, and this elevation causes ME, which leads to the loss of vision in a patient. Thus, intravitreal anti-VEGF administration is recommended for the treatment of BVO-ME and has become the mainstream treatment choice in recent years.

Regrettably, most of previous reports on intravitreal anti-VEGF treatment for BVO-ME disclosed only short-term outcomes (less than 3 years). On the other hand, there have been only a few reports about long-term outcomes (more than 3 years) [3, 4]. In this study, we investigated long-term visual outcomes treated with intravitreal anti-VEGF therapy for BVO-ME. Furthermore, we aimed to clarify valuable predictors for the long-term visual prognosis treated with anti-VEGF treatment for BVO-ME based on retinal structure by OCT, without consideration of the specific type of anti-VEGF drugs used.

## Methods

Patients with BVO-ME were retrospectively recruited based on clinical records in Kansai Medical University Hospital from June 2012 to March 2022. This retrospective study had Institutional Review Board approval from Kansai Medical University (#2,021,104) and was conducted in accordance with the tenets of the Declaration of Helsinki. In our hospital, the management options for BVO-ME are as follows: 1) no treatment (only observation), 2) vitrectomy, 3) administration of drugs, such as steroids or inhibitors of VEGF, and 4) laser photocoagulation. This study enrolled patients with vision loss who received intravitreal injection of anti-VEGF for BVO-ME. Inclusion criteria were the treatment naïve patients who are experiencing their first occurrence of BVO-ME and received intravitreal injection of anti-VEGF as their first treatment for BVO-ME. They must be followed up for at least 36 months at Kansai Medical University. Exclusion criteria were the patients with ocular disease other than RVO, including cataracts, or the patients who had been previously treated for BVO-ME. As a result, 47 eyes from 45 patients with BVO-ME were assessed. Each patient underwent a complete ophthalmologic examination, including assessment of best-corrected visual acuity (BCVA) using a 5-m Landolt chart, assessment of intraocular pressure, slit-lamp biomicroscopy, and fundus examination. BCVA was measured as the decimal visual acuity, then converted to the logarithm of the minimum angle of resolution (log MAR) for statistical analysis. ME was evaluated by color fundus photography (CFP; Topcon TRC50DX; Topcon, Tokyo, Japan), an ultra-wide-field scanning laser ophthalmoscope (California P200DTx; Optos, England), and spectral-domain OCT (SD-OCT; Heidelberg Spectralis OCT®, Heidelberg Engineering GmbH). Retinal ischemic was evaluated by fundus fluorescein angiography (FFA; Topcon TRC50DX, Topcon and California P200DTx; Optos, England).

In this study, patients were divided into two groups according to BCVA at the final visit: Group A (32 patients), with the final decimal BCVA of 0.7 or higher (meaning a good response to treatment) and Group B (15 patients), with the final decimal BCVA of lower than 0.7 (meaning a poor response to treatment). According to the above classification, we examined the 5 parameters listed below. To identify associations between VA and clinical features, we set the BCVA value of 0.7 to separate groups. With regards to BCVA in driver’s license in the world, for example, the drivers must have 0.5 of BCVA in United States of America and EU. In Japan, most strict BCVA (0.7) must be required in the world. Therefore, we decided to use a stricter cutoff the final decimal BCVA value of 0.7, which is the visual acuity requirement for obtaining a driver's license in Japan [5].

All patients with BVO-ME were treated with intravitreal injections of anti-VEGF (ranibizumab or aflibercept). No other type of anti-VEGF drugs applied in this study. The choice of anti-VEGF drug (ranibizumab or aflibercept) was decided by the treating physicians. In this study, the effectiveness of these 2 drugs was not investigated separately. Anti-VEGF therapy was administered immediately (within 7 days after diagnosis) for all patients in this study. After the first treatment, patients were followed-up approximately every 4 weeks (no more than 6 weeks) and at irregular intervals, as decided by the treating physicians. Patients were repeatedly treated by the regimen of 1 + *pro re nata* (PRN). Retreatment criteria were persistence or recurrence of ME, which sometimes accompanies serous retinal detachment (SRD) as detected by OCT, with a central retinal thickness (CRT) of more than 300 μm on the OCT image, or as having a visual acuity decrease of more than 2 ranks [6]. In addition, at 6 months after initial injection, and subsequently every 6 months after initial injection, all patients were evaluated for the ischemic subtype using fundus fluorescein angiogram (FFA) image. All BVO patients with retinal ischemia were performed by focal laser.

### Change in BCVA

BCVA was evaluated before treatment (baseline) and at 6 months after initial injection, 1 year after initial injection, 2 years after initial injection, 3 years after initial injection, and the final visit.

### Change in CRT

CRT was defined as the distance from the inner surface of the inner limiting membrane to the outer portion of the hyperreflective line, which corresponds to the retinal pigment epithelium. CRT was measured at baseline, 6 months after initial injection, 1 year after initial injection, 2 years after initial injection, 3 years after initial injection, and the final visit.

### Macular retinal structure using SD-OCT

We investigated the macular retinal structure using SD-OCT by assessing the following: (1) ellipsoid zone (EZ) integrity; (2) the presence of a foveal bulge; and (3) the presence of SRD. EZ integrity and the presence of a foveal bulge were evaluated at 1 month after the initial injection, 6 months after injection, and the final visit. We evaluated these structures at 1 month after initial injection rather than at baseline to avoid ill-visualization due to overlying retinal hemorrhages or ME. The presence of SRD was evaluated at baseline, and we evaluated its recurrence at 6 months after initial injection and at the final visit.

### Number of anti-VEGF treatments

The number of anti-VEGF treatments was counted at 6 months after initial injection, within the 1st year after initial injection, within the 2nd year after initial injection, and within the 3rd year after initial injection.

### Analysis of fundus fluorescein angiogram

Retinal ischemia was measured using a single FFA image at 6 months after initial injection, and subsequently every 6 months after initial injection. Retinal ischemic subtype was defined as more than 10 disc areas of retinal capillary nonperfusion based on FFA [3].

### Statistical evaluations

Statistical calculations were performed using JMP software (SAS Institute, Inc., Cary, NC, USA). Data are presented as means ± SD or medians (first quartile to third quartile) depending on whether the data had a normal distribution, as assessed by Bartlett’s test. Statistical analyses on the number of anti-VEGF intravitreal injections were performed by Student’s *t* test. Statistical analyses on the changes in BCVA and CRT were performed by the non-parametric Wilcoxon signed-rank test. Statistical analyses on the ratio that the number of eyes which EZ was intact within the total eyes, the ratio that the number of eyes which a foveal bulge was observed within the total eyes, the ratio that the number of eyes which the SRD/recurrent SRD was observed within the total eyes, and the number of eyes that displayed a retinal ischemic within the total eyes were performed by Fisher’s exact test. *P* values < 0.05 were considered statistically significant.

## Results

Baseline demographic characteristics of 47 eyes from 45 patients (23 male, 22 female) with BVO-ME are shown in Table 1. The mean age at initial presentation was 65.7 ± 8.5 years and the mean follow-up period from initial to final visit was 64.38 ± 15.07 (range, 38–100) months.

**Table 1:** Demographics and imaging characteristics

Eyes (*n*)	47
Age (mean ± SD^a^)	65.7 ± 8.5
Sex (male / female, *n*)	23/24
Follow-up period, months (mean ± SD, range)	64.38 ± 15.07 (38–100)
Log MAR^b^ BCVA^c^ (median, IQRs^d^)	
Baseline	0.301 (− 0.080–1.523)
6 months after injection	0.046 (− 0.176–1.097)
1 year after injection	0.097 (− 0.301–0.558)
2 years after injection	0.046 (− 0.301–0.824)
3 years after injection	0.046 (− 0.301–0.824)
Final visit	0.097 (− 0.176–1.523)
CRT^e^, μm (median, IQRs)	
Baseline	525.5 (309–966)
6 months after injection	284.0 (214–814)
1 year after injection	307.0 (216–825)
2 years after injection	327.0 (215–598)
3 years after injection	333.5 (245–803)
Final visit	304.5 (213–631)

^a^*SD* standard error

^b^*log MAR* logarithm of the minimum angle of resolution

^c^*BCVA* best-corrected visual acuity

^d^*IQRs* interquartile ranges

^e^*CRT* central retinal thickness

### Change in BCVA

**Fig. 1:**
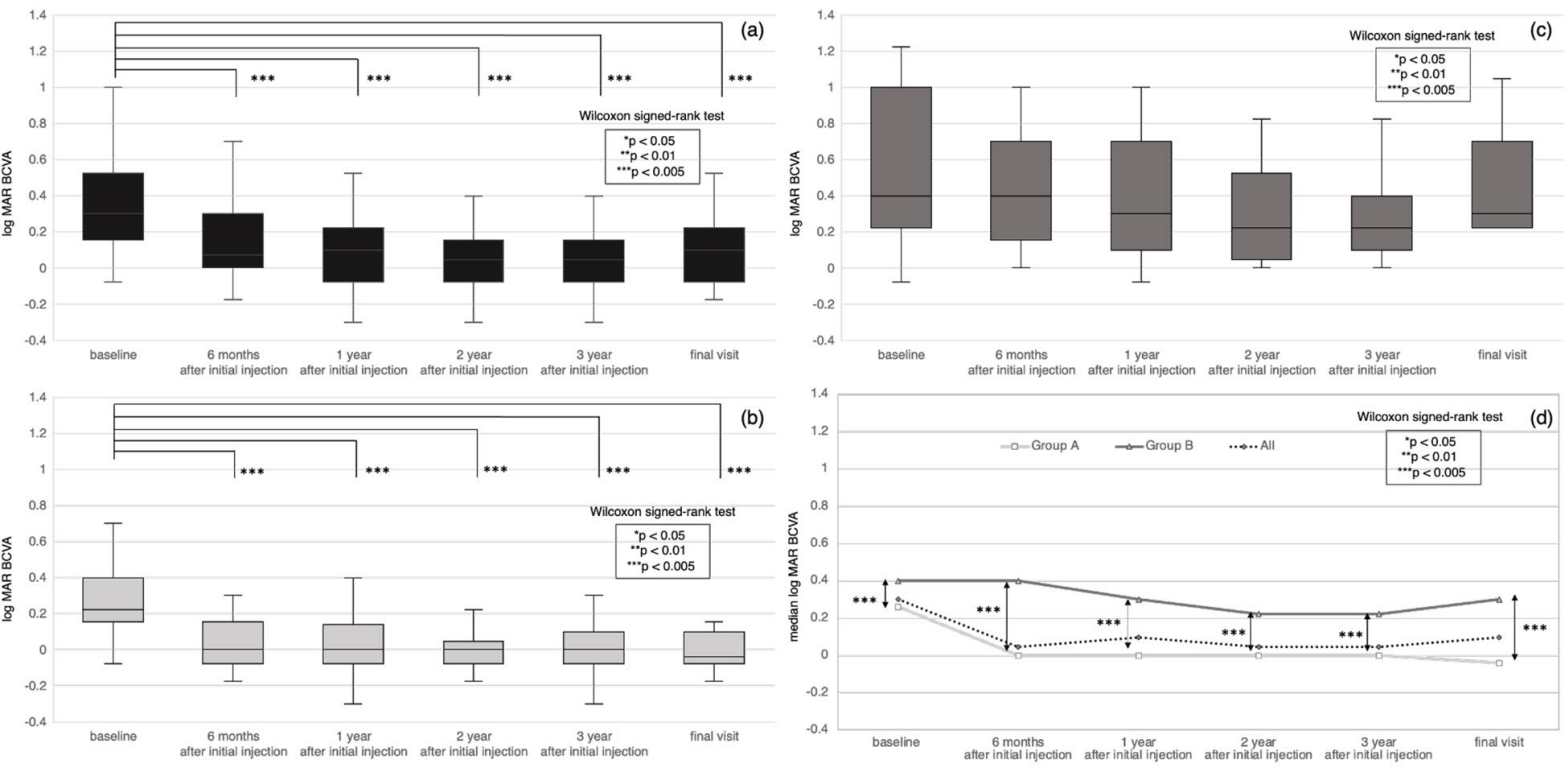
Change in BCVA. Patients were divided into two groups according to best-corrected visual acuity (BCVA) at the final visit: Group A (≥ 0.7) and Group B (< 0.7). **a** BCVA was significantly improved in all eyes after the initial injection of anti-VEGF treatment. Patients who had an improved BCVA by 6 months after the initial injection tended to maintain the improvement up to the final visit. **b** BCVA was significantly improved in Group A after the initial injection. Patients who had an improved BCVA by 6 months after the initial injection tended to maintain the improvement up to the final visit. **c** BCVA was not significantly improved in Group B. **d** BCVA was significantly greater in Group A than in those in Group B at all observation periods

The median BCVA (log MAR value) for all eyes is shown in Table 1 and Fig. 1(a). BCVA was significantly improved after the initial injection (*p* < 0.0001). Patients who had an improved BCVA by 6 months after the initial injection tended to maintain the improvement up to the final visit.

The median BCVA values for Groups A and B are shown in Table 2 and Fig. 1(b, c, d). BCVA was significantly improved in Group A after the initial injection (*p* < 0.0001). Patients who had an improved BCVA by 6 months after the initial injection in Group A tended to maintain the improvement up to the final visit 2.

**Table 2:** Comparison of the clinical characteristics between Group A and Group B

	**Group A**^**a**^	**Group B**^**b**^	***p* Value**
Total eyes (*n*)	32	15	
Follow-up period, months (mean ± SD^c^, range)	62.1 ± 15.6	69.33 ± 13.0	
Log MAR^d^ BCVA^e^ (median, IQRs^f^)			
Baseline	0.261 (− 0.079–1.523)	0.400 (− 0.079–1.222)	0.0283*
6 months after injection	0.000 (− 0.176–1.097)	0.400 (0.000–1.000)	< 0.0001*
1 year after injection	0.000 (− 0.301–0.523)	0.222 (− 0.079–1.000)	0.0025*
2 years after injection	0.000 (− 0.301–0.301)	0.222 (− 0.000–0.824)	0.0001*
3 years after injection	0.000 (− 0.301–0.400)	0.222 (0.000–0.824)	< 0.0001*
Final visit	− 0.040 (− 0.176–0.155)	0.301 (0.222–1.523)	< 0.0001*
CRT^g^, μm (median, IQRs)			
Baseline	511.0 (309–966)	565.0 (340–956)	0.7196
6 months after injection	283.5 (229–705)	291.0 (214–814)	0.5880
1 year after injection	298.0 (247–715)	404.0 (216–825)	0.0651
2 years after injection	315.0 (229–598)	365.0 (215–535)	0.4004
3 years after injection	328.0 (245–803)	378.0 (247–526)	0.1022
Final visit	286.0 (213–472)	344.0 (296–631)	0.0017*

^*^Symbol means *p* Value is < 0.05 that is considered statistically significant (Wilcoxon signed-rank test)

^a^Group A, the decimal final BCVA with 0.7 or higher

^b^Group B, the decimal final BCVA with lower than 0.7

^c^*SD* standard error

^d^*log MAR* logarithm of the minimum angle of resolution

^e^*BCVA* best-corrected visual acuity

^f^*IQRs* interquartile ranges

^g^*CRT* central retinal thickness

The BCVA in Group A was significantly greater than in Group B at all observation periods (baseline, *p* = 0.0283; 1 year, *p* = 0.0025; and 6 months, 2 years, 3 years, and final visit, *p* < 0.0001).

### Change in CRT

**Fig. 2:**
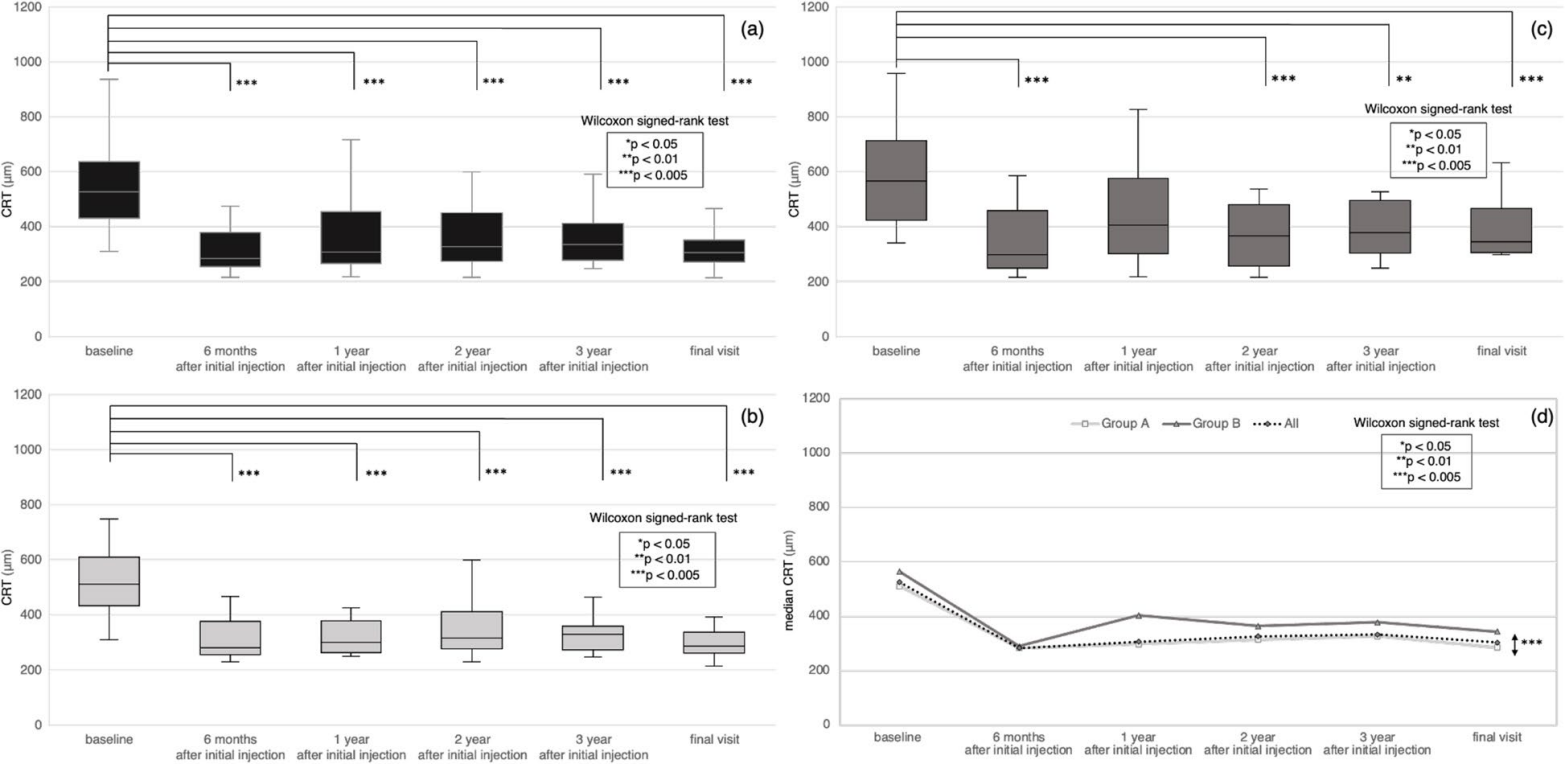
Change in CRT. Patients were divided into two groups according to best-corrected visual acuity (BCVA) at the final visit: Group A (≥ 0.7) and Group B (< 0.7). **a** Central retinal thickness (CRT) was significantly reduced in all eyes after the initial injection. Patients who had a reduced CRT by 6 months after the initial injection tended to maintain the reduction up to the final visit. **b** CRT was significantly reduced in Group A after the initial injection. Patients who had a reduced CRT by 6 months after the initial injection tended to maintain the reduction up to the final visit. **c** CRT was significantly reduced in Group B after the initial injection, except at the 1-year mark. Patients who had a reduced CRT by 6 months after the initial injection tended to maintain the reduction up to the final visit. **d** CRT at the final visit was significantly reduced in Group A compared to that in Group B

The median CRT for all eyes is shown in Table 1 and Fig. 2(a). CRT was significantly reduced after the initial injection (*p* < 0.0001). Patients who had a reduced CRT by 6 months after the initial injection tended to maintain the reduction up to the final visit.

The median CRTs in Groups A and B are shown in Table 2 and Fig. 2(b, c, d). CRT was significantly reduced in Group A after the initial injection (*p* < 0.0001). In Group B, CRT was also significantly reduced after the initial injection, except for the timepoint at 1 year after initial injection (baseline vs 6 months, *p* = 0.0013; baseline vs 1 year, *p* = 0.0779; baseline vs 2 years, *p* = 0.0030; baseline vs 3 years, *p* = 0.0064; baseline vs final visit, *p* = 0.0018).Patients who had a reduced CRT by 6 months after the initial injection in Group A and B tended to maintain the reduction up to the final visit.

CRT at the final visit in Group A was significantly lower compared to that in Group B (*p* = 0.0017).

### Macular retinal structure using SD-OCT

The number of eyes that displayed a clear layer of EZ, foveal bulge, and (recurrent) SRD in Groups A and B are shown in Table 3. The ratio that the number of eyes which EZ was intact was significantly higher in Group A than Group B (*p* = 0.0004). The ratio that the number of eyes which a foveal bulge was observed in Group A was significantly higher than that in Group B (*p* = 0.0002).

**Table 3:** Comparison of the macular retinal structure using SD-OCT^a^ between Group A^b^ and Group B^c^

	**Group A**	**Group B**	***p* Value**
Total eyes (*n*)	32	15	
The EZ^d^ band was intact (*n*, %)			
1 month after injection	20 (62.5%)	1 (6.67%)	0.0004*
6 months after injection	17 (53.1%)	0 (0.0%)	0.0002*
Final visit	17 (53.1%)	0 (0.0%)	0.0002*
A foveal bulge was observed (*n*, %)			
1 month after injection	20 (62.5%)	0 (0.0%)	< 0.0001*
6 months after injection	20 (62.5%)	0 (0.0%)	< 0.0001*
Final visit	17 (53.1%)	0 (0.0%)	0.0002*
SRD^e^ was observed at baseline (*n*, %)	22 (68.8%)	8 (53.3%)	0.3441
The recurrence SRD was observed (*n*, %)			
by 6 months after injection	15 (46.9%)	9 (60%)	0.5343
by the final visit	17 (53.1%)	13 (87%)	0.0485*

^*^Symbol means *p* Value is < 0.05 that is considered statistically significant (Fisher’s exact test)

^a^*SD-OCT* spectral-domain optical coherence tomography

^b^Group A, the decimal final BCVA with 0.7 or higher

^c^Group B, the decimal final BCVA with lower than 0.7

^d^*EZ* ellipsoid zone

^e^*SRD* serous retinal detachment

There was no significant difference between Groups A and B in the ratio the number of eyes which SRD was observed at baseline. There was also no significant difference between Groups A and B in the ratio that the number of eyes which recurrence SRD was observed by 6 months after the initial injection (*p* > 0.05). In contrast, the ratio the number of eyes which recurrence SRD was observed by the final visit in Group A was significantly lower than that in Group B (*p* = 0.0485).

### Number of anti-VEGF intravitreal injections

**Fig. 3:**
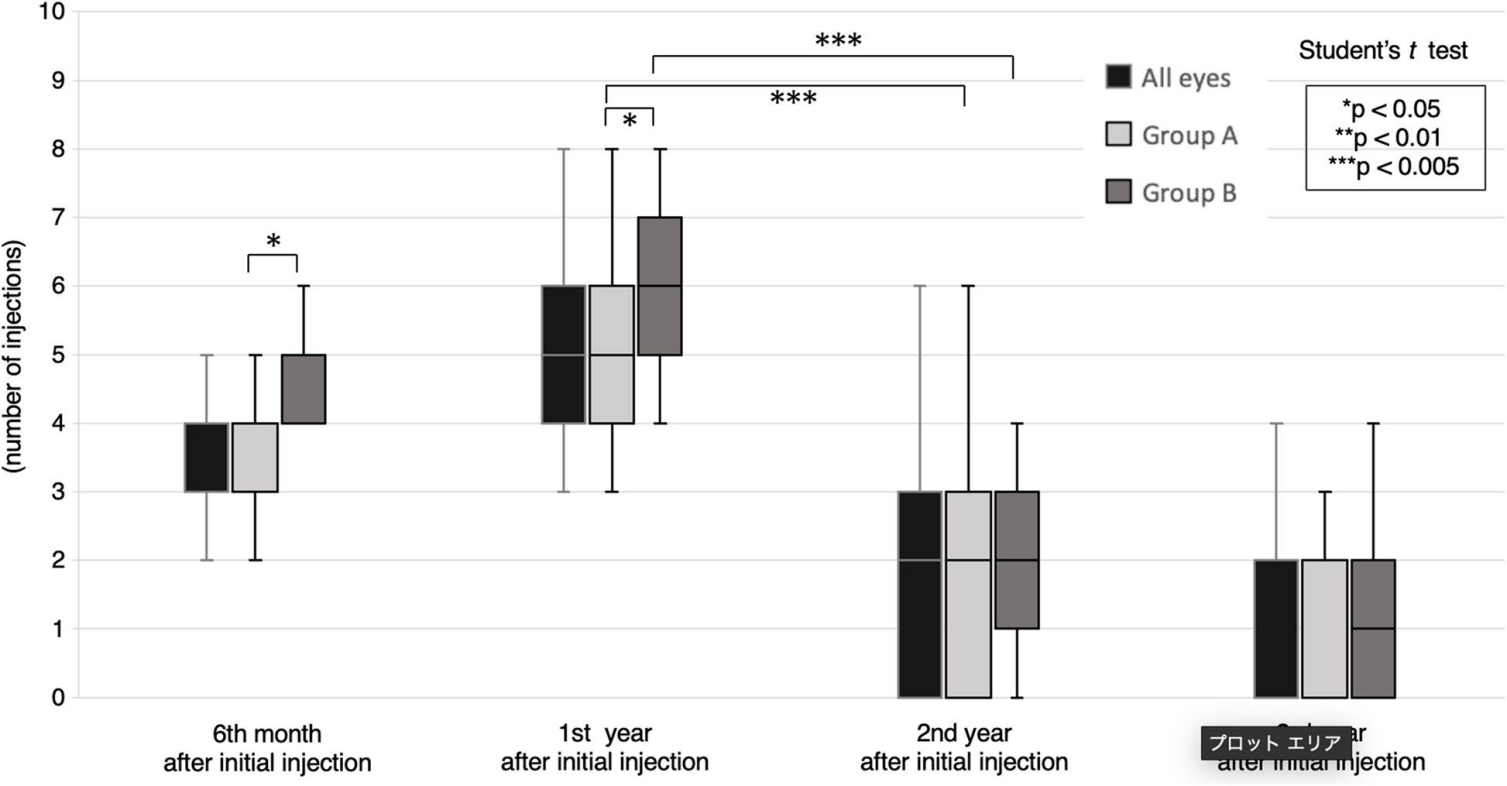
Number of anti-VEGF treatments. Patients were divided into two groups according to best-corrected visual acuity (BCVA) at the final visit: Group A (≥ 0.7) and Group B (< 0.7). The number of injections in the 2nd year after the initial injection was significantly lower than in the 1st year after the initial injection in all groups. There was also a significant difference between Groups A and B in the number of injections in the 6th month and in the 1st year after initial injection

The mean number of intravitreal anti-VEGF injections in all eyes, as well as split by Group A and Group B, are shown in Fig. 3. The number of anti-VEGF intravitreal injections in the 2nd year after initial injection was significantly lower than that in the 1st year (all eyes, Group A, and Group B, *p* < 0.0001). In addition, the number of injections in the 3rd year after the initial injection was significantly lower than that in the 2nd year (all eyes, *p* = 0.0013; Group A, *p* < 0.0001; Group B, *p* = 0.0399).

The number of anti-VEGF intravitreal injections in Group A by the 6th month and the 1st year after initial injection were significantly lower than those in Group B (6th month, *p* = 0.0008; 1st year, *p* = 0.0207). In contrast, no significant difference was observed between Group A and B in the 2nd or 3rd year after initial injection (2nd year, *p* = 0.8034; 3rd year, *p* = 0.8008).

### Analysis of fundus fluorescein angiogram

The patients in this study were divided into 2 groups between the retinal ischemic or non-ischemic BVO. The number of eyes that displayed a retinal ischemic in Groups A and B are shown in Table 4. There was no significant difference between Groups A and B in the ratio of ischemic BVO. BVO patients with retinal ischemia were performed by focal laser. No cases with neovascularization were observed.

**Table 4:** Comparison of the ischemic subtype between Group A^a^ and Group B^b^

	**Group A**^**a**^	**Group B**^**b**^	***p*** **Value**^**c**^
Total eyes (*n*)	32	15	
The ischemic type^d^ (*n*, %)	8 (25%)	5 (33.3%)	0.7278

^a^Group A, the decimal final BCVA with 0.7 or higher

^b^Group B, the decimal final BCVA with lower than 0.7

^c^*p* Value is < 0.05 that is considered statistically significant (Fisher’s exact test)

^d^Retinal ischemic subtype was defined as more than 10 disc areas of retinal capillary nonperfusion based on FFA

## Discussion

Although many clinical trials and studies have been conducted on intravitreal anti-VEGF administration for BVO-ME, most had only a short-term follow-up (less than 3 years) [7]. These studies showed that baseline BCVA, CRT, the integrity of the EZ layer and foveal bulge, and the presence of SRD are predictors of post-treatment visual outcomes. However, to date, limited data exist on long-term (more than 3 years) outcomes. Based on the anti-VEGF therapy followed by a PRN regimen for BVO-ME, we investigated to reveal valuable predictors for the long-term visual prognosis for BVO-ME from our long-term (average of 64 months follow-up) study. This study demonstrated that baseline BCVA, the integrity of the EZ layer, and the presence of a foveal bulge were significant predictors of post-treatment visual acuity in the long-term, as well as in the short-term, as shown in other studies. In contrast to other studies, this study showed that CRT was not a useful predictor based on long-term observation. Although most studies that have a short-term follow-up showed that the presence of SRD negatively affected visual prognosis, our long-term analysis showed that the presence of SRD at initial presentation did not affect visual prognosis. Still, the presence of recurrent SRD did predict a poor visual prognosis.

To identify associations between visual acuity and clinical features, we classified patients with BVO-ME into 2 groups based on their final BCVA and investigated several parameters for visual prognosis (i.e., Group A [good response to treatment], with a final decimal BCVA of 0.7 or higher; Group B [poor response to treatment] with a final decimal BCVA of lower than 0.7). Table 1 showed significant improvements in BCVA and reductions in CRT in all eyes after the initial injection. Short-term reports [2, 6, 7] have shown a close correlation between 1) final BCVA and baseline BCVA and 2) final BCVA and baseline CRT. In our study, Table 2 showed, there were significant differences between Groups A and B for median BCVA through all observation periods. In contrast, there were no significant differences between Groups A and B for the median CRT at any observation timepoint, except at the final visit. Therefore, baseline BCVA is a useful predictor for long-term visual prognosis, as well as short-term prognosis, as previously shown in other short term studies. In contrast, our study showed that CRT was not a useful predictor for the long term visual prognosis. Interestingly, Group A showed a significant improvement in BCVA in the initial 6 months after treatment start. Moreover, the improved BCVA in Group A was maintained until the final visit. In comparison, Group B did not show the same tendency as Group A. Hasegawa T et al. also reported that an improvement in BCVA was not observed later than 7 months after the initial injection, although there was an improvement during the initial 6 months after treatment start [8]. From the results of Hasegawa’s report and ours, BCVA did not show a remarkable improvement more than 7 months after initial treatment. Therefore, BCVA at 6 months after initial injection is a useful predictor for long-term visual prognosis.

According to the BRAVO and CRUISE studies, improved BCVA and reduced CRT correlated with better visual prognosis in BVO-ME [9–11]. However, in the current study, a reduction of CRT did not correlate with better visual prognosis. Although the BRAVO and CRUISE studies did not analyze the retinal outer segment shape, other reports have studied OCT parameters other than CRT as predictors of post-treatment visual acuity in patients with BVO-ME [7, 12]. For example, Rupak R et al. suggested that the EZ band and foveal bulge must be intact to gain better visual outcomes. We investigated macular retinal structure (EZ, foveal bulge, SRD) using OCT in Groups A and B, as shown in Table 3. Our study evaluated EZ integrity and the presence of a foveal bulge at 1 month, 6 months after initial injection, and at the final visit. In our study, the ratio that the number of eyes which intact EZ and the presence of a foveal bulge were observed in Group A was significantly higher than that in Group B. We suppose this may be because the EZ band and foveal bulge in Group B had already been damaged at 1 month after the initial injection and never repaired by repeated treatments. Therefore, we believe that severe damage to photoreceptor cells during the ME leads to substantial defects in the outer segments (OS) of the photoreceptor cells, resulting in a lack of the EZ band. Hasegawa T et al. suggested that the foveal bulge seen in OCT images could be related to the morphologic maturation of the photoreceptor OS, which is characterized by an increase in foveal photoreceptor OS density and elongation of foveal photoreceptor. In other words, the lack of a foveal bulge was induced by shortening of the photoreceptor OS length and a decrease in foveal photoreceptor OS density. In addition, even with foveal cystoid spaces in the retina, good visual function may be maintained only if the foveal photoreceptor layer, especially its outer aspect, is preserved. Therefore, similar to Hasegawa’s report, our study showed that integrity of the EZ band and an intact foveal bulge are good predictors of visual function for BVO-ME in the long-term. Moreover, our patients who had severe foveal photoreceptor damage never improved their BCVA in our long-term study. Based on these results, we believe it is very important to protect photoreceptor cells while treating BVO-ME.

Peter A Campochiaro et al. reported that a worsened BCVA in BVO-ME patients was induced by damage to photoreceptors from retinal ischemia and recurrent retinal edema. Photoreceptor damage is more likely to occur with prolonged and/or repeated severe retinal edema. From short-term studies, patients who had SRD at initial presentation tended to have worse visual acuity during the follow-up [13]. Broken retinal capillaries lead to sub-retinal fluid, thickening the retina. Then, Müller cell cones are stretched perpendicularly along the walls of the foveal cystoid spaces. Extended Müller cell cones break the external limiting membrane (ELM) barrier, resulting in SRD [14]. However, even though the presence of SRD is believed to be the most important factor for evaluating visual prognosis in patients with BVO-ME, our long-term study showed that SRD at initial presentation did not affect final visual acuity. Nonetheless, the presence of a higher recurrent SRD ratio led to poor visual acuity (Table 3). According to previous reports, VA and CRT can be improved by anti-VEGF treatment in patients with BVO-ME with or without SRD [13, 15]. In addition, a more marked improvement of macular structure was achieved in patients with SRD than in those without SRD. These findings may be explained by the strong association of inflammatory factors and extensive morphological changes with the occurrence and/or recurrence of SRD, thus patients with SRD have better improvement of macular structure by anti-VEGF treatment [13]. Based on previous reports and the current study, we suggest that if SRD is observed only at the first presentation, it does not necessarily predict poor visual prognosis because anti-VEGF treatment can alleviate damage to foveal photoreceptors in the outer segment by restoring the ELM barrier and improving macular structure and function [16]. In comparison, the presence of recurrent SRD causes further damage to the retina, resulting in poor visual outcomes. Therefore, it is important to introduce anti-VEGF treatment at appropriate timing, with attention to the presence of recurrent SRD, to better optimize visual outcomes.

Unlike in the case of AMD, there is no standard regimen for RVO-ME with anti-VEGF agents. In the BRAVO trial, patients received monthly intravitreal ranibizumab (IVR) in the first 6 months, and additional injections during the subsequent 6 months if the patient met the prespecified criteria (6 + PRN regimen). In contrast, the BRIGHTER trial confirmed the efficacy of a less frequent treatment regimen (3 + PRN regimen), where patients received monthly IVR until the VA was stable for 3 consecutive months, and if VA did not change after the last monthly treatment, the next re-treatment was warranted only when VA decreased and if the decrease was due to disease activity in the opinion of the investigator. Considering the relatively good final-visit BCVA in this study (0.097 log MAR unit, which is equivalent to 80 ETDRS letters), we believe the BCVA at the final visit here was comparable to that in the BRIGHTER study (75 ETDRS letters) [17]. Moreover, our 1 + PRN regimen over the course of 2 years maintained the BCVA with fewer IVR injections compared with the PRN regimen over 2 years of IVR injections used in the BRIGHTER study (7.1 ± 2.41 vs 11.4 ± 5.81 injections, respectively). Therefore, 1 + PRN regimen of anti-VEGF injections for BVO-ME could lead to an improved BCVA and be useful in clinical practice.

Similar to the RETAIN study, which reported that 50% of patients with BRVO were treated by anti-VEGF injections for over 4 years, our study also demonstrated that many patients receive long-term anti-VEGF injections (64.38 ± 15.07 [range, 38–100] months) to maintain or improve their visual function. Therefore, long-term observation with proper treatment is essential for better visual outcomes.

In this study, there was no correlation between retinal ischemia and final-visit BCVA. Although short-term study [18] reported that the number of anti-VEGF intravitreal injections for ischemic type BVO is higher than those of non-ischemic type BVO, there is no significant difference in visual prognosis between ischemic type BVO and non-ischemic type BVO. In our study as well, there was no significant difference in the ratio of ischemic type BVO between Group A and Group B. Therefore, it was observed that ischemic subtype did not affect long-term visual prognosis. In addition, no cases with neovascularization were observed. The early intervention of anti-VEGF therapy or appropriate focal laser treatment has been effective in preventing vision loss caused by neovascularization. Based on the results of the ZIPANGU study did not demonstrate the clinical utility of adding focal/grid laser treatment to ranibizumab monotherapy [6], combination therapy of anti-VEGF and focal/grid laser for the RVO-ME was not employed in this study. However, recent reports [19, 20] have indicated short-term benefits of combining laser therapy with anti-VEGF therapy, such as reductions in subretinal fluid and a decrease in the frequency of anti-VEGF intravitreal injections. This suggests that the combination of laser therapy could potentially become a beneficial treatment option.

There were some limitations in this study. First, it was a retrospective study design with no control group and small sample size. Second, the choice of anti-VEGF drug, including ranibizumab or aflibercept, was decided by the treating physicians. The effectiveness of these 2 drugs was not investigated separately. Third, patients who had less than three years of follow-up were excluded from this study. This may have introduced a selection bias, if the excluded patients were good responders to anti-VEGF treatment.

## Conclusions

This study demonstrated that the integrity of the EZ band and an intact foveal bulge (signifying intact photoreceptor cells) were significant predictors of post-treatment visual acuity in the long-term and short-term. In comparison, we found that recurrent SRD led to poor visual acuity in this long-term study, even if patients with BVO-ME had a good BCVA in the short-term. Therefore, this study showed that recurrent SRD is an important indicator for poor visual prognosis during long-term observation.

## Data Availability

All data generated or analyzed during this study are included in this published article.
